# Trivalent influenza vaccination randomized control trial of pregnant women and adverse fetal outcomes

**DOI:** 10.1016/j.vaccine.2019.07.024

**Published:** 2019-08-23

**Authors:** Eric A.F. Simões, Marta C. Nunes, Phyllis Carosone-Link, Richard Madimabe, Justin R. Ortiz, Kathleen M. Neuzil, Keith P. Klugman, Clare L. Cutland, Shabir A. Madhi, for the Matflu Team

**Affiliations:** a Medical Research Council: Respiratory and Meningeal Pathogens Research Unit, Faculty of Health Sciences, University of the Witwatersrand, York Road, Parktown, Johannesburg 2193, South Africa; b Colorado School of Public Health, Center for Global Health, University of Colorado School of Medicine, Aurora Colorado, 13123 E. 16^th^ Ave., B055 Aurora, CO, United States; c University of Colorado Denver, Dept. of Pediatric Infectious Diseases, 13123 E. 16^th^ Ave, B055 Aurora, CO, United States; d Department of Science and Technology/National Research Foundation: Vaccine Preventable Diseases, University of the Witwatersrand, York Road, Parktown, Johannesburg 2193, South Africa; e University of Maryland Center for Vaccine Development, 685 W Baltimore St, Baltimore, MD, USA; f Bill and Melinda Gates Foundation, 500 5th Ave N, Seattle, WA, USA; g National Institute for Communicable Diseases: a division of National Health Laboratory Service, Centre for Vaccines and Immunology, 1 Modderfontein Road, Sandringham, Johannesburg, South Africa

**Keywords:** Low birth weight, Prematurity, Randomized controlled trial, Small for gestational age

## Abstract

**Introduction:**

The purpose of this study was to evaluate the association of influenza vaccine during pregnancy and adverse fetal outcomes. Preventing fetal death, low birth weight, small for gestational age birth and preterm birth are important potential effects of antenatal maternal influenza immunization for which there are conflicting data.

**Materials and methods:**

A double-blind, randomized, placebo-controlled clinical trial of trivalent inactivated influenza vaccine was conducted in South Africa from March 2011 until after the 2012 influenza season when the infants born had reached the age of 24 weeks. Mothers were administered the vaccine or placebo during pregnancy at a gestation of 20 to 36 weeks. A comparison of rates of fetal death, low birth weight, small for gestational age birth, and preterm birth, between vaccinated and placebo groups was made. Fetal outcome differences between the groups were measured using Student’s t-tests, vaccine efficacy with 95% confidence intervals, and Poisson regression for incidence rates. All analyses except fetal death excluded mothers who were administered vaccine or placebo after 34 weeks gestational age.

**Results:**

There were 2116 HIV-uninfected pregnant women age 18 to 38 years in the trial; 2005 infants were born to mothers where vaccine or placebo had been administered ≥ 14 days prior to delivery, and there were 6 miscarriages and 23 stillbirths. There was no significant vaccine efficacy (with [95% confidence interval]) on fetal death (−21.2% [−150.8, 41.4]), low birth weight (−11.1% [−42.3, 12.5]), small for gestational age birth (−9.9% [−35.6, 11.0]), or preterm birth (−21.3% [−60.5, 8.3]). Neither was vaccine efficacy demonstrated when the analysis was restricted to infants of mothers who were exposed to an influenza season (1832 outcomes available).

**Conclusion:**

We did not find a beneficial effect of trivalent inactivated influenza vaccine during pregnancy on adverse fetal outcomes.

## Highlights

• A two-year randomized controlled trial of seasonal IIV3.
• Over 2000 mothers in South Africa, from March 2011 until post 2012 influenza season.
• Birth outcomes investigated were fetal death, SGA, birth weight and prematurity.
• Vaccine demonstrated no appreciable impact of maternal IIV3 immunization.

## 1 Introduction

Antenatal maternal immunization with influenza vaccine can prevent influenza illness in the mother and her infant in the first few months of life [1], [2], and it can also provide benefits to the fetus [3]. A post-hoc analysis of a randomized controlled trial of influenza vaccine vs pneumococcal polysaccharide vaccine, conducted in Bangladesh, first suggested that influenza vaccine given to pregnant women could have a protective effect against small for gestational age (SGA) births and low birth weight (LBW) among a subset of infants born during the influenza season [4]. However the limitations of Mother’s Gift Trial included that it was underpowered, it did not have predetermined analyses, and its active comparator arm necessitated larger, more robust studies, which were placebo controlled with pre-specified outcomes of adequate sample size. Subsequently, a number of observational studies from the Americas and Europe, including over 350,000 pregnancies showed improved outcomes for one, or the other, or both of these outcomes or prematurity [5], [6], [7], [8]. Two of these studies [4], [5], demonstrated this impact specifically among infants born during the influenza season. A 2016 WHO Consultation on influenza vaccination association with adverse birth events have highlighted methodological limitations in the published observational literature [9]. Many published studies may not have fully adjusted for important differences in the vaccinated and unvaccinated cohorts [10], [6], [7], [8]. Subsequently, three recent, large population-based studies from Denmark [11], [12], Italy [13] and the US, [14] that used propensity scores to control for confounding, found no influence on fetal death, spontaneous abortion or stillbirth [12] and either of the rates of prematurity or small size for gestational age [11], [13], [14].

The resolution of the potential effect of the influenza vaccine on the fetus is of considerable importance in developing countries [15], where neonatal mortality accounts for 45% of under-5 mortality, a significant proportion of which is linked to premature birth/low birth weight and its complications [16]. Thus even small increases in the birth weight or decreases in the rate of premature birth could have a substantial impact on neonatal and infant mortality, regardless of the ultimate cause of death. Recognizing the potential beneficial effect of antenatal maternal influenza immunization, The Bill & Melinda Gates Foundation funded three large randomized placebo-controlled trials, in Mali [17], Nepal [18] and South Africa [1], [19]. While no effect of the vaccines was demonstrated on prematurity rates in the Nepalese [18] and South African [1] trials, there was a 14% reduction in low birth weight demonstrated only in the Nepalese trial; the main difference between vaccinated and placebo groups was 42·1g [95% CI 8·2 – 76·0]. The primary goal of our study in South Africa was to determine the efficacy of trivalent inactivated influenza vaccine against laboratory-confirmed infant and maternal influenza in HIV-uninfected mother-infant dyads [1]. A secondary objective of our study was to determine the effect of maternal influenza immunization on fetal outcomes.

## 2 Materials and methods

### 2.1 Study design

A double-blind, randomized, placebo-controlled clinical trial of trivalent inactivated influenza vaccine (IIV3; Vaxigrip®, Sanofi Pasteur) was conducted in Soweto, South Africa during 2 consecutive influenza seasons (2011 and 2012). Enrollment started on March 3, 2011, for the first cohort and on March 6, 2012, in the second year. Eligibility criteria included maternal ages 18 to 38 years, estimated gestational age between 20 and 36 weeks, and absence of certain medical conditions. Complete inclusion and exclusion criteria, vaccine characteristics and subject demographics are described elsewhere [1]. Follow-ups were completed after the influenza seasons when the infants born had reached the age of 24 weeks [1]. The studies included 2116 HIV-uninfected pregnant women age 18 to 38 years, administered influenza vaccine or placebo between 20 and 36 weeks of gestation.

Methods for estimating vaccine efficacy of IIV3 versus placebo in protecting mothers and their infants from contracting influenza illness, comparison of seroconversion rates, and safety outcomes are discussed elsewhere [1], [20]. The purpose of this secondary analysis was to evaluate the efficacy of IIV3 vaccination during pregnancy on adverse fetal outcomes (positive or negative), namely fetal death, prematurity, SGA, and LBW. To these ends, two subgroups and their infants were studied: (1) All mothers enrolled before or during an influenza season with delivery before, during or after an influenza season, and (2) Mothers pregnant and at risk of influenza during the influenza season only.

The study (ClinicalTrial.gov number NCT01306669) was approved by the Human Research Ethics Committee of the University of the Witwatersrand (HREC number: 101106) and conducted in accordance with Good Clinical Practice guidelines. Signed, written informed consent was obtained from all participants.

### 2.2 Participants

Full cohort. All Mothers Enrolled Before or During an Influenza Season with Delivery Before, During or After an Influenza Season. This analysis included mothers who were enrolled in the study, whose fetal outcomes were known, and who had been administered vaccine or placebo a minimum of 14 days prior to delivery. The time between mother’s enrollment and delivery was used to compute the person-years (PY) of enrollment for incidence rate analysis and was counted whether or not that time period fell within an influenza season. All subjects who fit these criteria were included in the fetal death analyses. All other analyses included only live-born infants. This analysis is a modified intention-to-treat vaccine efficacy analysis. Further, live-born infants of mothers who were administered vaccine or placebo after 34 weeks gestational age (wGA) were excluded from the live-born analyses.

Influenza season gestation Subgroup. Mothers at Risk During Influenza Season Only. The South African influenza seasons were defined using the National Institute for Communicable Diseases surveillance data. (2011: 16 May2011-06 Nov2011; 2012: 21 May2012-14 Oct2012) [1], [21] Mothers who were enrolled and administered vaccine or placebo during an influenza season prior to delivery, whose fetal outcomes were known, *and* who had been administered vaccine or placebo a minimum of 14 days prior to delivery, were included in this subgroup. For incidence rate analysis, only the PY of enrollment until delivery that fell within an influenza season were counted towards the mother’s PY. This subgroup only included the outcomes of fetuses gestating during the influenza season and therefore were potentially at risk for maternal influenza virus infection associated outcomes. As per the previous cohort, live-born infants of mothers who were administered vaccine or placebo after 34 wGA were excluded from the live-born analyses.

### 2.3 Randomisation, masking, and procedures

Study subjects were randomized shortly after they consented to participate in the study and eligibility had been confirmed. Except for the statistician and pharmacist, all study personnel and participants were blinded to the computer-generated, randomly assigned [1:1 ratio] designation of mothers who received either IIV3 or placebo. Participants received either 0·5 ml of the influenza vaccine with the southern hemisphere composition for 2011 and 2012 [Vaxigrip, Sanofi Pasteur] in the active arm or an identical-looking placebo of 0·5 ml of 0·9% normal saline, administered into the deltoid muscle by study nurses.

### 2.4 Sample size requirements

Sample size requirement was computed *a priori* for the RCT study regarding vaccine efficacy versus contracting influenza [1]. A post-hoc power analysis was computed to determine the detectable difference in infant birth weight between the intervention groups with 80% power and alpha of 5%, given the existing sample size, and common standard deviation of birth weight, utilizing a Student’s *t*-test.

### 2.5 Outcomes

Fetal outcomes were classified as: fetal death from miscarriages, spontaneous abortion of pregnancy occurring after 20 wGA, and stillbirths defined as a fetal death after 28 weeks gestation [22]. Gestational age was determined by the Ballard method [23] and recorded by the attending medical provider during delivery for all preterm (≤37^6/7^ weeks) births. For term births (>37^6/7^ weeks), the GA was computed as days elapsed between the enrollment visit and the birth, plus GA (in days) that was determined at enrollment. Gestational age of the mother at enrollment was determined using a hierarchy of methods which included, by order of priority, fetal ultrasound when available, the last menstrual period of the mother, and physical examination by palpation of fundal height [1]. Birth weight was classified as normal (NBW) [>2500 g], and low (LBW) [<2500 g]) [24]. SGA was defined as < 10th percentile in the INTERGROWTH-21st Consortium published international standards for newborn baby size centile charts [25].

Study data were collected and managed using REDCap [26] electronic data capture tools hosted at the University of Colorado Denver, CO, USA.

### 2.6 Statistical analysis

All statistical analyses were pre-specified and were performed using SAS version 9·4 (SAS Institute Inc., Cary NC, USA). Graphs were created using SPSS v.22 (IBM Corp, Armonk, NY, USA). Data were analysed from the viewpoints of efficacy and safety, as these outcomes could be viewed from both viewpoints. We defined efficacy using simple vaccine efficacy (VE) proportions, however granting that safety examinations require a person time denominator for comparison across studies, we have also analyzed data (safety analyses) using a person time denominator. Comparisons between means were performed using a two-tailed, two-sample Student’s *t*-test.

Vaccine efficacy was calculated using the formula 100 × (1 − I_v_/I_p_), where I_v_ = incidence rate in the vaccinated-group and I_p_ = incidence rate in placebo-group; 95% confidence intervals (95% CI) were constructed [27], and differences between the intervention group rates tested for significance [28].

For the safety analyses, Incidence Rate Ratios (IRR) were computed using the formula (I_v_/I_p_). Incidence rates were computed per 1000 PY for these analyses. The associated 95% CI and *P*-values were computed using PROC GENMOD, using an exact approximation to the Poisson distribution.

The endpoints of this analysis included fetal death, preterm birth, LBW and SGA, as defined above, gestational age and birth weight. Since maternal influenza infection could potentially affect the fetus at different gestational ages and its prevention might thus affect the fetus differentially, we examined more finely defined outcomes in the secondary efficacy and safety outcomes (Supplementary results).

## 3 Results

**Fig. 1:**
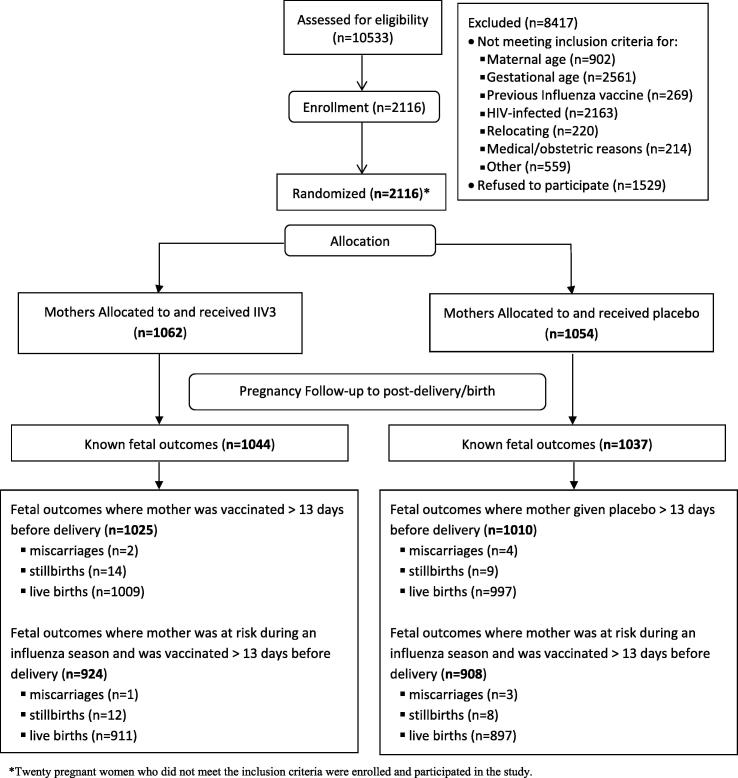
Study population, subgroups, and reasons for exclusion for this vaccine study and results for fetal outcomes.

A flow diagram (Fig. 1) depicts the study population, subgroups, and reasons for exclusion for this vaccine study.

*Sample Size Requirements.* After excluding mothers who were administered vaccine or placebo after 34 wGA, we were left with 1884 (cohort 1) and 1737 (cohort 2) liveborn infants with a known birth weight. Given these N, we are able to detect a difference in birth weight of 39 g with 69% power (cohort 1) and 65% power (cohort 2), using a 2-tailed *t*-test analysis. Using a 1-tailed test, we are able to detect a difference of 40 g with 79% power (cohort 1) and 76% power (cohort 2).

### 3.1 Outcomes

There were 2081 mothers with known fetal outcomes; of these, 2035 mothers met the criterion of being vaccinated at least 14 days prior to delivery (N = 1025 in IIV3-group and N = 1010 in placebo-group). There were 29 fetal deaths distributed among the two study groups with VE of −21·2% (95% Confidence Intervals (CI): −150·8, 41·4) (Table 1). The mean GA at birth was significantly higher in the placebo-group (mean ± SD = 38.9w ± 2.7w) compared to the IIV3-group (mean ± SD = 38.6 w ± 2.7w; *P* = 0.025), with a concomitant non-significant 39 g difference in birth weight (x̅_P_ ± SD = 3032 g ± 518 g in IIV3-group vs. 3071 g ± 500 g in placebo-group; *P* = 0.094). VE fetal outcome measures (preterm birth, LBW and SGA) and safety for the full cohort are presented in (Table 1, Table 2, Supplemental Table 1). Finer breakdowns of GA (term, post term and preterm), and birth weight (appropriate for gestational age, SGA and LBW) and their combinations for VE are presented in Supplemental Table 1.

**Table 1:** The efficacy of IIV3-vaccination of pregnant women in preventing fetal death, preterm birth, low birth weight, and small for gestational age infants for mothers who were administered vaccine or placebo on or before 34 weeks gestation.

	IIV3 Vaccine	Placebo		
Outcome	N^a^/Total N (%)	N^a^/Total N (%)	VE (95%CI)	*P*
*Over The Duration Of Mother’s Enrollment Until Delivery*				
Fetal Death b	16/1025 (1.6)	13/1010 (1.3)	−21.2 (−150.8, 41.4)	0.60
Preterm Birth c	100/958 (10.4)	80/930 (8.6)	−21.3 (−60.5, 8.3)	0.17
Low Birth Weight c,d,^e^	123/956 (12.9)	107/928 (11.5)	−11.1 (−42.3, 12.5)	0.38
Small for Gestational Age (SGA) c,e,f,^g^	156/955 (16.3)	138/928 (14.9)	−9.9 (−35.6, 11.0)	0.38

*In Women At Risk During The Influenza Seasons*				
Fetal Death b	13/924 (1.4)	11/908 (1.2)	−16.1 (−157.9, 47.7)	0.71
Preterm Birth c	77/885 (8.7)	61/856 (7.1)	−17.2 (−14.4, 40.1)	0.22
Low Birth Weight c,d,^e^	105/883 (11.9)	85/854 (10.0)	−27.7 (−67.3, 2.5)	0.20
Small for Gestational Age (SGA) c,e,f,^g^	149/882 (16.9)	130/854 (15.2)	−9.9 (−11.8, 27.4)	0.34

a N = number of fetal outcomes.

b N = total number of subjects without regard to their gestational stage at vaccine or placebo administration.

c Using only live births; excluded 1 subject whose gestational age of 21 weeks at birth was incongruent with her birth weight of 3185 g.

d LBW is < 2500 g.

e Birth weight missing for two subjects who were excluded.

f <10th percentile weight for GA.

g Sex was missing for one subject and thus percent weight for gestational age could not be computed for that subject.

**Table 2:** The safety of IIV3-vaccination of pregnant women in preventing fetal death, preterm births, low birth weight, and small for gestational age outcomes for mothers who were administered vaccine or placebo on or before 34 weeks gestation.

Outcome	IIV3 Vaccine		Placebo		IRR (95%CI)	*P*
	N a	Rate a	N a	Rate a		
*Over The Duration Of Mother’s Enrollment Until Delivery*						
Fetal Death b	16	69.1	13	57.1	1.21 (0.58, 2.52)	0.61
Preterm Birth c	100	446.3	80	365.1	1.22 (0.91, 1.64)	0.18
Low Birth Weight c,^d^	123	549.0	107	488.3	1.12 (0.87, 1.46)	0.38
Small for Gestational Age (SGA) c,e,^f^	156	697.0	138	629.7	1.11 (0.88, 1.39)	0.38

*In Women At Risk During The Influenza Seasons*						
Fetal Death b	13	58.8	11	50.8	1.16 (0.52, 2.58)	0.72
Preterm Birth c	77	356.8	61	290.2	1.23 (0.88, 1.72)	0.23
Low Birth Weight c,^d^	105	486.6	85	404.3	1.20 (0.90, 1.60)	0.20
Small for Gestational Age (SGA) c,e,^f^	149	691.3	130	618.4	1.12 (0.88, 1.41)	0.35

a N = number of fetal outcomes; Rate per 1000 person-years of mother’s study participation until delivery.

b N = total number of subjects without regard to their gestational stage at vaccine or placebo administration.

c Using only live births; excluded 1 subject whose gestational age of 21 weeks at birth was incongruent with her birth weight of 3185 g.

d LBW is < 2500 g.

e <10th percentile weight for GA.

f Sex was missing for one subject and thus percent weight for gestational age could not be computed for that subject.

**Fig. 2:**
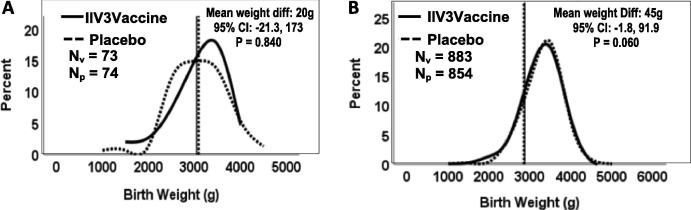
Birth weight for infants (N) born prior to the influenza seasons (Panel A), and those born during or following the influenza seasons (Panel B), by intervention status (P = Placebo; V = IIV3).

There were 1832 known fetal outcomes whose mothers were at risk during an influenza season (N = 924 in IIV3-group and N = 908 in placebo-group). There were 24 fetal deaths and 1808 live births. GA was statistically greater in the placebo versus the vaccinated group (mean ± SD = 39·0 ± 2·6w, mean ± SD = 38·8 ± 2·6w, respectively; *P* = 0.048). Mean birth weight was lower in the IIV3 group (x̅_v_ = 3046 g, ±510 g; x̅_p_ = 3091 g ± 484 in the placebo group; *P* = 0.060) but only approached significance; birth weight differences are graphically illustrated (Fig. 2). The percentage of LBW infants was not statistically different in the two groups (12.5% in the IIV3-group vs. 11.5% in the placebo-group; *P* = 0.38; Table 1). There were no significant findings of VE (Table 1) and no significant differences between incidence rates for any of the fetal outcomes (Table 2).

The birth weight of infants born prior to an influenza season did not differ by mother’s intervention status (Fig. 2, panel A). The infants born during or following an influenza season had consistently higher birth weights in the placebo group (Fig. 2, panel B), albeit not statistically significant. Mean person-years was compared between vaccine and control groups, separately for those infants born prior to and during/after the influenza season. There were no significant differences in follow-up per person-years (*P* = 0.242 prior to season; *P* = 0.655 during/after influenza season).

## 4 Discussion

Our study, which is one of 3 recent placebo-controlled trials of IIV3 given to pregnant women [1], [17], [18], found no effects of the vaccine on predefined fetal outcomes, as measured by the birth weights and birth gestational ages and the rates of fetal death, prematurity, low birth weight and small size for gestational age. We posited that if the vaccine was to have a biological effect, it could be explained by prevention of influenza virus infection in the mothers prior to delivery. Hence we examined all of these outcomes when mothers were pregnant and potentially exposed to circulating influenza virus, once again demonstrating no appreciable effect (subgroup 2) [Table 1]. We next examined the possibility that protection against exposure to influenza before versus during/after the influenza season would have had a differential impact on birth weight, and again found no significant associations (Fig. 2). It is unlikely that vaccine would have made a difference prior to the influenza season (Fig. 2, Panel A). The non-significant difference between vaccine and placebo for birth weight during/after the influenza season, is consistent with the absence of VE for birth weight-related variables in Table 1 and Supplemental Table 1.

In fact, we found a slight, though non-significant, decrease in the birth weight of infants in the vaccinated group [−40 g mean difference], overall or restricted to infants of mothers who were potentially exposed to influenza *in utero* [Fig. 2B], as well as a non-significant increase in fetal deaths among IIV3-vaccinated mothers. Consistently there was a slight, though non-significant, imbalance in birth weights in the same direction when we examined pregnancy during the influenza season as an exposure. We point this out only as a cautionary word and suggest this observation be explored carefully in larger studies of vaccine safety databases such as the Vaccine Safety Datalink study group or similar vaccine safety groups in Europe.

Our results differ substantially from the first randomized controlled trial from Bangladesh [4]. What might be the reasons? This was a placebo-controlled trial, whereas the Bangladeshi trial had an active control, pneumococcal polysaccharide vaccine (PPV). While the authors of the Bangladeshi trial, ascribed the result of increased birth weight and lower rates of SGA to influenza vaccine, it is possible that the outcomes in the PPV active control arm were due to a detrimental effect of the active control. We subgrouped our outcomes based on fetal exposure to potential maternal influenza, during the influenza season whereas the Bangladeshi study subgrouped outcomes by birth during the influenza season. Our analyses are predicated on the potential biologically plausible hypotheses that (a) maternal influenza infection has an impact on the fetus resulting in fetal death, LBW, SGA and prematurity and conversely that (b) protecting mothers from influenza via IIV3 would prevent these adverse fetal outcomes. Biologically it is unclear why this impact of maternal IIV3-immunization would occur only in children born during the influenza season; however, we conducted an ad-hoc analysis of our data to address the impact during the influenza season. We did not find any significant difference in birth weight, prematurity or SGA when we examined these outcomes for infants born within and out of the influenza seasons (data not shown). In Bangladesh, the influenza season is longer [29] and hence the effect of the vaccine might have been dependent on a potentially longer period of exposure to the influenza season. The longer potential period of exposure in Bangladesh might, however, be countered with a shorter period of protection since mothers were immunized after 27 weeks gestation as opposed to our 20 weeks. A fourth explanation could be that the underlying birth weight of infants in Bangladesh was lower than those in our South African infants. However, the mean birth weight of the infants in the PPV (control) arm in the Bangladeshi study was 3027 g, which is comparable to the 3074 g in our study. If these explanations were valid, one would not expect to see any effect of the vaccine in North America and Europe, as was suggested in several observational studies [10], [5], [6], [7], [8]. The interpretation of these studies however should be tempered with others that robustly controlled for bias, in Denmark, Italy and North America [11], [12], [13], [14]. These studies and a *meta*-analysis [30], did not find any impact of influenza vaccination of mothers on fetal outcomes either. Supporting our findings, are several studies that did not specifically examine birth weight and prematurity as primary outcomes, since they were done primarily for safety [31], [32], [33].

Given the global importance of preventing LBW, prematurity and small size for gestational age with a simple intervention, we urge caution in generalizing our findings, in light of the results of the other randomized controlled trials in Mali [17] and Nepal [18]. Both of those studies immunized mothers year round, as necessitated by the almost year-round seasonality of influenza in the study sites [17], [18]. In all three studies from Mali [17], Nepal [18] and in ours, there was no significant impact on prematurity, or low birth weight in the protocol prescribed analysis of data. However, the pooled analysis of two years of the Nepalese study [18], demonstrated a 40-gram increase in birth weight. This translated to a 15% reduction in low birth weight rate [risk ratio 0·85, 95% CI 0·75 – 0·97] in the IIV3 vaccinated group compared to control. This translated to less than the 200 g increase in the Bangladeshi study when influenza was circulating. In the Nepalese study the mean birth weight of babies in the placebo group was 2761 G compared to 3075 G in our study and 3015 in the Malian study, and there was a much higher rate of LBW (27%) than in our study (11%) or the Malian study (8·2%). The mean maternal BMI in the Nepalese study was 20·9 compared to a median of 27·4 in ours. Perhaps the impact of maternal influenza vaccinations might be of more importance in countries with lower birthweights than in Mali or South Africa.

Of note, none of the three trials showed an impact on premature birth. It is possible that this was because all three trials were underpowered to show an impact as suggested by Hutcheon et al. [34] in a recent mathematical modeling exercise using the epidemiological literature. A post-hoc sample size analysis was performed to determine whether there were sufficient data to detect vaccine efficacy against birth weight. While our study sample size of 2035, appears to be slightly underpowered to answer this question, the combined numbers in the three trials (3693 in Nepal and 4193 in Mali with our 2035 total = 9921) could potentially be powered to answer this question in a planned *meta*-analysis.

Given the CIs, this neutral study can rule out a vaccine efficacy of 12% for LBW, 8% for preterm birth, 11% for SGA, and 41% for fetal death (Table 1). From these data, it can be concluded that the vaccine has no clinically meaningful efficacy with respect to LBW, preterm birth, SGA, and fetal death. Viewed from a safety perspective and given the CIs around the RRs, the study can rule out increased risks of more than 46% for LBW, 39% for SGA, 64% for preterm birth, 152% for fetal death (Table 2).

## 5 Conclusions

Despite a significant reduction in maternal influenza infections [1], our 2-year randomized controlled trial of seasonal IIV3 in over 2000 mothers in South Africa, demonstrated no appreciable impact of maternal IIV3 immunization on SGA, birth weight or prematurity, either directly or indirectly. Prevention of influenza in the mother and their babies is the primary rationale for providing influenza vaccine to pregnant women. While effects on birth outcomes would be an additional benefit, we did not find evidence in this population of HIV-uninfected South African women.

## Declaration of Competing Interest

Author declares that there is no conflicts of interest.
