# Supplementary material for: Trivalent influenza vaccination randomized control trial of pregnant women and adverse fetal outcomes
Source: Vaccine. 2019 Aug 23;37(36):5397–403. doi: 10.1016/j.vaccine.2019.07.024 (PMC6694200; doi:10.1016/j.vaccine.2019.07.024)
Supplement: Supplementary data 1 [file mmc1.docx]

| \| **Supplemental Table 1. The efficacy of IIV3-vaccination of pregnant women in preventing preterm birth, LBW, SGA, and combinations of these outcomes for mothers who were administered vaccine or placebo on or before 34 weeks gestation: Over The Duration Of Mother’s Enrollment Until Delivery** \| \| \| \| \| \| --- \| --- \| --- \| --- \| --- \| \|  \| **IIV3 Vaccine** \| **Placebo** \|  \|  \| \| **Outcome** \| **N^a^/Total N (%)** \| **N^a^/Total N (%)** \| **VE (95%CI)** \| ***P*** \| \| **Term^b^** \| 790/958 (82.5) \| 774/930 (83.2) \| 0.9 (-3.2, 4.9) \| 0.66 \| \| **Post Term^b^** \| 68/958 (7.1) \| 76/930 (8.2) \| 13.1 (-19.0, 36.6) \| 0.38 \| \| **Birth Weight >= 2500 G^b^** \| 833/956 (87.1) \| 821/928 (88.5) \| 1.5 (-1.9, 4.8) \| 0.38 \| \| **Appropriate or large birth weight for gestational age^b, c^** \| 799/955 (83.4) \| 790/928 (85.1) \| 1.7 (-2.2, 5.5) \| 0.38 \| \| **Preterm LBW^b^** \| 51/956 (5.3) \| 43/928 (4.6) \| -15.1 (-71.0, 22.5) \| 0.48 \| \| **Preterm not LBW^b^** \| 49/956 (5.1) \| 37/928 (4.0) \| -29.0 (-95.1, 15.3) \| 0.24 \| \| **Term LBW^b^** \| 72/956 (7.5) \| 64/928 (6.9) \| -9.2 (-51.5, 21.1) \| 0.59 \| \| **Term not LBW^b^** \| 784/956 (82.0) \| 784/928 (84.5) \| 2.9 (-1.1, 6.8) \| 0.15 \| \| **Preterm SGA^b, c^** \| 10/955 (1.0) \| 8/928 (0.8) \| -21.5 (-206, 51.9) \| 0.68 \| \| **Preterm not SGA^b, c^** \| 90/955 (9.4) \| 72/928 (7.8) \| -21.5 (-63.4, 9.7) \| 0.20 \| \| **Term SGA^b, c^** \| 146/955 (15.1) \| 130/928 (14.0) \| -9.1 (-35.8, 12.30) \| 0.43 \| \| **Term not SGA^b, c^** \| 709/955 (74.2) \| 718/928 (77.4 ) \| 4.1 (-1.0, 8.8) \| 0.11 \| |
| --- | --- | --- | --- | --- | --- | --- | --- | --- | --- | --- | --- | --- | --- | --- | --- | --- | --- | --- | --- | --- | --- | --- | --- | --- | --- | --- | --- | --- | --- | --- | --- | --- | --- | --- | --- | --- | --- | --- | --- | --- | --- | --- | --- | --- | --- | --- | --- | --- | --- | --- | --- | --- | --- | --- | --- | --- | --- | --- | --- | --- | --- | --- | --- | --- | --- | --- | --- | --- | --- | --- | --- | --- | --- | --- | --- |
| ^a^ N = number of fetal outcomes  ^b^ Using only live births; one subject’s gestational age at birth of 21 weeks was incongruent with her birth weight of 3185 g, and was excluded.  ^c^ Sex was missing for one subject and thus this subject’s percent weight for gestational age could not be computed; |
