# Supplementary material for: Trivalent influenza vaccination randomized control trial of pregnant women and adverse fetal outcomes
Source: Vaccine. 2019 Aug 23;37(36):5397–403. doi: 10.1016/j.vaccine.2019.07.024 (PMC6694200; doi:10.1016/j.vaccine.2019.07.024)
Supplement: Supplementary data 2 [file mmc2.docx]

| **Supplemental Table 2. The efficacy of IIV3-vaccination of pregnant women in preventing preterm birth, VLBW, LBW, SGA, and Combinations of these outcomes for mothers who were administered vaccine or placebo on or before 34 weeks gestation: In Women At Risk During The Influenza Seasons** | | | | | | |
| --- | --- | --- | --- | --- | --- | --- |
|  | **IIV3 Vaccine** | | **Placebo** | |  |  |
| **Outcome** | **N^a^ (%)** | **Total N^a^** | **N^a^ (%)** | **Total N^a^** | **VE (95%CI)** | ***P*** |
| **Term^b^** | 740 (82.6) | 885 | 721 (84.2) | 856 | 0.7 (-3.4, 4.7) | 0.73 |
| **Post Term^b^** | 68 (7.7) | 885 | 74 (8.6) | 856 | 11.1 (-21.9, 35.2) | 0.46 |
| **Birth Weight >= 2500 G^b^** | 778 (88.1) | 883 | 769 (90.1) | 854 | 2.0 (-1.1, 5.3) | 0.20 |
| **Appropriate or large birth weight for gestational age^b, c^** | 733 (83.1) | 882 | 724 (84.8) | 854 | 2.0 (-2.2, 5.9) | 0.34 |
| **Preterm LBW^b^** | 38 (4.3) | 883 | 30 (3.5) | 854 | -22.5 (-95.9, 23.4) | 0.39 |
| **Preterm not LBW^b^** | 39 (4.4) | 883 | 31 (3.6) | 854 | -21.7 (-93.2, 23.4) | 0.40 |
| **Term LBW^b^** | 67 (7.6) | 883 | 54 (6.3) | 854 | -20.0 (-69.6, 15.1) | 0.35 |
| **Term not LBW^b^** | 739 (83.7) | 883 | 738 (86.4) | 854 | 3.2 (-0.7, 6.9) | 0.11 |
| **Preterm SGA^b, c^** | 8 (0.9) | 882 | 7 (0.8) | 854 | -10.7 (-204, 307) | 0.84 |
| **Preterm not SGA^b, c^** | 69 (7.8) | 882 | 54 (6.3) | 854 | -23.7 (-74.4, 12.2) | 0.22 |
| **Term SGA^b, c^** | 141 (16.0) | 882 | 123 (14.4) | 854 | -11.0 (-38.7, 11.2) | 0.36 |
| **Term not SGA^b, c^** | 664 (75.3) | 882 | 670 (78.5) | 854 | 4.0 (-1.03, 8.9) | 0.12 |
| ^a^ N = number of fetal outcomes  ^b^ Using only live births; one subject’s gestational age at birth of 21 weeks was incongruent with her birth weight of 3185 g, and was excluded.  ^c^ Sex was missing for one subject and thus this subject’s percent weight for gestational age could not be computed; | | | | | | |
