# Supplementary figures and images for: Trivalent influenza vaccination randomized control trial of pregnant women and adverse fetal outcomes
Source: Vaccine. 2019 Aug 23;37(36):5397–403. doi: 10.1016/j.vaccine.2019.07.024 (PMC6694200; doi:10.1016/j.vaccine.2019.07.024)

| Figure 2. Panel A |
| --- |
| 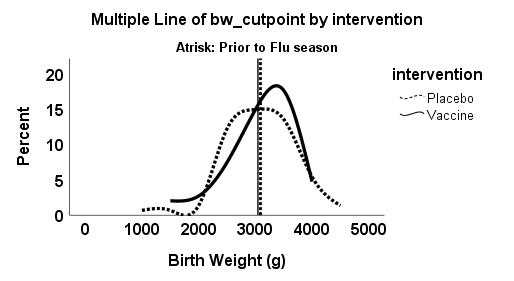 |
| Figure 2. Panel B |
| 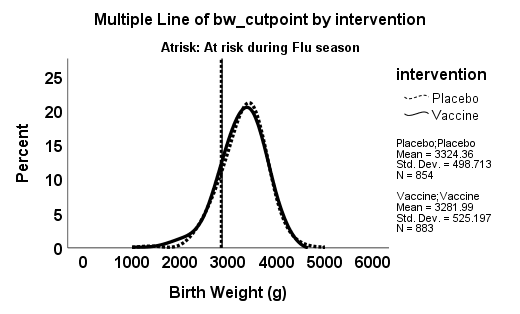 |

Supplement: Supplementary data 3 [file mmc3.docx]
